# Functional outcomes following ileal pouch-anal anastomosis (IPAA) in older patients: a systematic review

**DOI:** 10.1007/s00384-015-2475-4

**Published:** 2016-01-12

**Authors:** Lisa Ramage, Sheng Qiu, Panagiotis Georgiou, Paris Tekkis, Emile Tan

**Affiliations:** Colorectal Surgery, Imperial College London, London, UK; Department of Surgery and Cancer, Chelsea and Westminster Hospital, 369 Fulham Road, London, SW10 9NH UK

**Keywords:** Ileal pouch-anal anastomosis (IPAA), Restorative proctocolectomy (RPC), Age, Postoperative function

## Abstract

**Aim:**

Ileal pouch-anal anastomosis (IPAA) is performed in ulcerative colitis or familial adenomatous polyposis with a view to restoration of GI continuity and prevention of permanent faecal diversion. Debate exists as to its safety in older patients. This review aims to assess functional outcomes and safety of restorative proctocolectomy (RPC) in older compared to younger patients.

**Methods:**

Literature search was performed for age-stratified studies which assessed functional outcomes of IPAA. Twelve papers were included overall. Patients were categorized into ‘older’ and ‘younger’ groups. Analysis was split into three separate parts: 1. Age cut-off of 50 ± 5 years (with sensitivity analysis); 2. Age cut-off of 65 ± years; 3. Long-term outcomes (>10 years).

**Results:**

With an age cut-off of 50 years (4327 versus 513 patients), complication rates were comparable with the exception of an increased rate of small-bowel obstruction in the younger patients (*p* = 0.034). At 1 year, 24-h stool frequency was significantly higher in the older patient group (*p* < 0.0001). Daytime (*p* < 0.0001) and night-time (*p* < 0.0001) incontinence rates were also significantly higher in older patients.

Overall, function deteriorated with time across all ages; however, after 10 years, there was no significant difference in incontinence rates between age groups.

Dehydration and electrolyte loss was a significant problem in patients over 65 (*p* < 0.0001).

Despite differences in postoperative function, quality of life was comparable between groups; however, only a few studies reported quality of life data.

**Conclusion:**

IPAA is safe in older patients, although treating clinicians should bear in mind the increased risk of dehydration. Postoperative function is worse in older patients, but seems to level out with time and does not appear to significantly impact on overall quality of life and patient satisfaction. Assessment for suitability for RPC should not be based on chronological age in isolation. It is imperative that the correct support is given to older patients with worsened postoperative function in order to maintain patient satisfaction and adequate quality of life.

## Introduction

Restorative proctocolectomy (RPC), also known as ileal pouch-anal anastomosis (IPAA) is typically performed in patients with familial adenomatous polyposis (FAP) or ulcerative colitis (UC). It can be performed either as a single-stage procedure or, in cases where emergency surgery has been required, as a completion proctectomy with pouch formation following emergency colectomy.

In the elderly, the majority of cases are performed in the context of UC which is non-responsive to maximal medical therapy or where there is evidence of associated dysplasia or malignancy [1]. Originally, the general consensus was that surgery in those over 50 should undergo proctocolectomy with formation of an end ileostomy, as the associated morbidity with IPAA was too high for older patients. However, several papers have documented similar outcomes with IPAA in terms of safety when compared to younger patients [2–4].

This review paper aims to examine the functional outcomes following IPAA in older versus younger patients. This will be performed through analysis of published literature undertaking age-related analysis of functional outcomes with IPAA.

## Methods

The systematic review was undertaken in accordance with the PRISMA (Preferred Reporting Items for Systematic Reviews and Meta-Analyses) guidelines. Two reviewers (LR and SQ) performed the literature search and data extraction independently. PubMed, Medline and Google scholar databases were searched for relevant articles. The following keywords and phrases were used in various combinations: age, function, outcome, elderly, ileoanal pouch, ileal anal-pouch anastomosis (IPAA) ileal pouch, restorative, proctocolectomy.

All articles identified within the initial search were screened for relevance and content, and their bibliographies searched for any additional relevant articles. Abstracts were initially screened by title and abstract content for relevance. In cases where relevance was uncertain, the papers were scanned for the relevant data.

The criteria for inclusion were as follows: 1. Articles considering postoperative functional outcomes with IPAA stratified according to patient age. 2. Ileoanal pouch formation for any disease condition (e.g. ulcerative colitis, familial adenomatous polyposis).

Exclusion criteria were as follows: 1. No discussion of at least one functional outcome of interest (see below), 2. Non-comparative papers, case reports and review papers. 3. Papers where age has been found to be a significant factor in outcome through multivariate analysis; however, no further information has been provided. 4. Papers published in an alternative language to English where translation was not available. 5. Papers where the ‘older’ age group cut-off was younger than 45 years.

All publications up to and including 1 February 2015 were considered. Major outcomes of interest were as follows:

1. Postoperative complications
2. Bowel frequency
3. Incontinence
4. Use of pads/medications
5. Quality of life (QoL) data
6. Patient satisfaction
7. Sexual function
8. Long-term data (>10 years)

Data extraction was undertaken independently by two of the authors (LR and SQ) using the pre-determined outcome measures. There were no discrepancies between them.

The following data points were extracted (where presented) from each paper:

Trial design, patient subgroups, sex ratio, diagnosis, indication for surgery, surgery performed, comorbidities, pouch configuration, use of covering ileostomy, handsewn or stapled anastomosis, length of follow-up, postoperative complications.

Functional outcomes: number of bowel movements per 24 h, number of movements/day, number of movements/night, pad usage, seepage, incontinence, urgency, deferral time, use of anti-diarrhoeals.

Anorectal physiology results: maximum/mean resting pressures, maximum/mean squeeze pressures, threshold and maximum tolerated volumes.

In addition to this, quality of life data, sexual function and patient satisfaction data were also retrieved where given.

### Statistical analysis

Due to the heterogeneity of presented data, it was often not feasible to perform statistical analysis. Means were combined to give weighted means for overall results. In cases where a median has been given, this was converted to a mean using the method described by Hozo and colleagues [5]. Student’s *t* test was used to compare continuous data, and chi-squared 2 × 2 contingency table was used to compare categorical data.

## Results

**Fig. 1:**
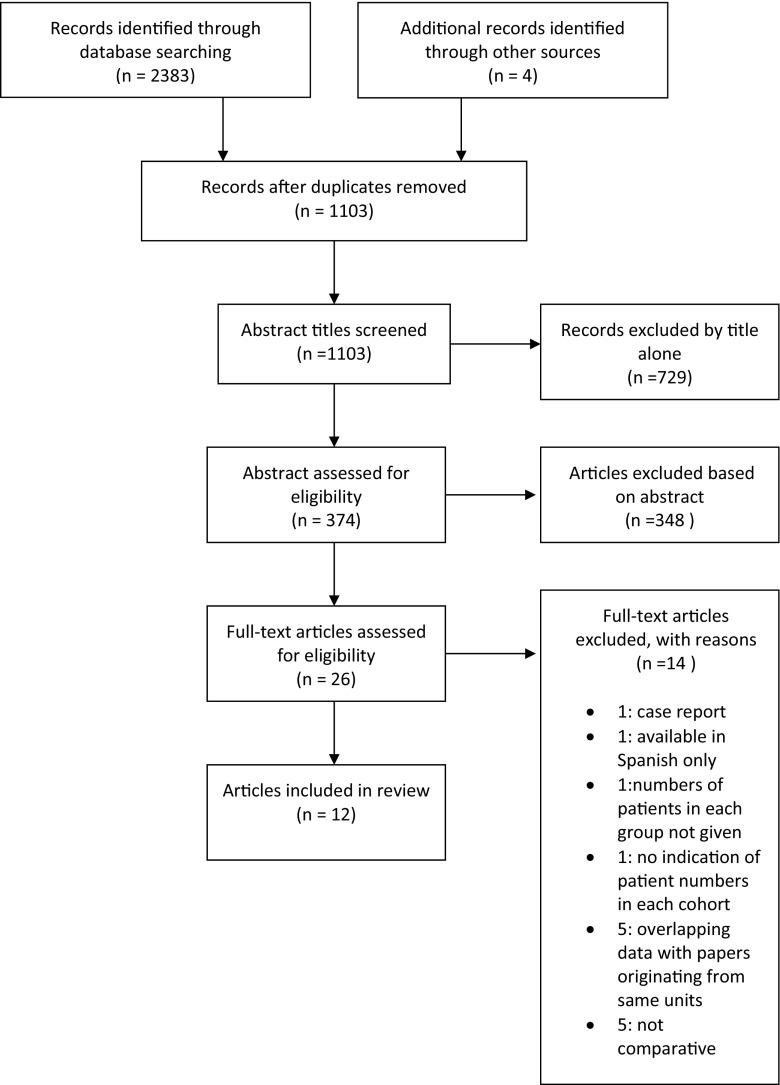
Flow chart of literature review

Figure 1 demonstrates the literature search results. Following exclusion of duplicates, 1103 abstracts were returned through the use of the search terms. A total of 729 papers were excluded based on title alone; 374 abstracts were reviewed for content and 348 were excluded.

A total of 26 papers were identified which contained data regarding age and functional outcomes with restorative proctocolectomy. Twenty-one papers were identified with documented comparative functional outcomes with IPAA across different age groups. One was excluded as this was a case report, and another was excluded as the article was available in Spanish only. The paper written by Pemberton et al. [6] was also excluded as this did not contain the numbers of patients in each sub-group; therefore, incorporation of data into the analysis was not possible.

Of the 18 papers left, five were found to be from the Cleveland Clinic Florida. Four papers contained overlapping patient data [7–10]. The paper by Ho et al. [10] was included as this was the most recent data; however, outcomes from Takao et al. [9] were included in discussion which were not featured in the more recent paper (anorectal physiology, patient satisfaction, incontinence scoring system).

Likewise, further three papers [10–13] originated from the same unit (Mayo clinic, Minnesota). The most recent data from Chapman et al. [13] was included in analysis.

Finally, there were three papers from the Cleveland Clinic in Ohio with overlapping patient data [14–16]. The 2003 paper written by Delaney and colleagues contained more short-term data, whereas the paper by Kiran et al. was concerned more with long-term functional results, with a follow-up of 15 years. Therefore, results published at the 1 year mark from Delaney et al. were used in the general analysis, with long-term results (at 15 years follow-up) taken from the paper by Kiran et al.

Overall, there were 12 papers included in this review. Study characteristics are given in Table 1.

**Table 1:** Study demographics

Study	Year	Design	From–to	Total no. of patients	Age categories (*n*)	Mean age	M:F	Disease	Disease duration (years)	Mean duration follow-up (months)
Pescatori	1990	Prospective database	1980–1989	207 (156 with functional data)	<45 (123) >45 (33)	Median 34 (8–67)	122 M:85 F	UC 141 FAP 65 Intractable constipation 1	ND	13.4 ± 16.5
Lewis	1993	Case matched	1986–1991	36	<50 (18) >50 (18)	31 years 55 years (median)	9 M:9 F 9 M:9 F	UC 36	ND	12 (6–15)^b^ 12 (3–24)^b^
Bauer	1996	Retrospective/prospective	1988–1996	392 (311with functional data)	<50 (263) >50 (51)	30.9 56.9	166 M:160 F 37 M:29 F	UC 392	ND	ND
Dayton	1996	Prospective database	1983–1996	455	<55 (423) >55 (32)	33 59	ND	UC 355 FAP 63 IC 34 Collagenous colitis 3	8.9 9.3	12
Tan	1997	Prospective	1983–1997	227	<50 (109) 50–60 (12) 60–70 (7) >70 (5)	ND	118 M:109 F	UC 168 CD 18 FAP 28 Functional 13	ND	72 (3–171)^b^ <70 62 (24–157)^b^ >70
Takao	1998	Prospective	ND	122	<40 (58) 40–60 (47) >60 (17)	ND	29 M:29 F 29 M:18 F 11 M:6 F	UC 122	ND	49 (25–177) 57 (24–137) 48 (25–71)
Delaney	2003	Prospective	1983–1999	1895 (1785 with functional data)	<45 (1323) 45–55 (277) 56–65 (146) >65 (93)	ND	1071 M:824 F	UC 1364 FAP 114 IC 303 CD 95 Cancer 6 Other 15	9.3 ± 8.8	4.6 ± 3.7
Chapman	2005	Prospective	1981–2000	2002	<45 (1866) 46-55 (249) >55 (65)	33.5	874 M:814 F 150 M:99 F 45 M:20 F	UC 1790 FAP 212	ND	10.1 ± 5.7
Ho	2005	Prospective database	1989–2001	330	<30 (79) 30–49 (148) 50–69 (86) >70 (17)	ND	29 M:50 F 60 M:88 F 41 M:45 F 5 M:12 F	UC 330	4.9 (0.6)^a^ 8.4 (0.6)^a^ 12.3 (1.4)^a^ 14.0 (3.7)^a^	ND
Kiran	2009	Retrospective, prospective database	1983–?	396	<35 (189) 35–55 (184) >55 (23)	36 (median)	210 M:186 F	UC 308 IC 46 CD 5 FAP 18	ND	17.1 (median)
Pinto	2009	Retrospective, prospective database	2001–2008	66	<65 (33) >65 (33)	36.9 ± 10.9 68.7 ± 3.8	11 M:22 F 11 M:22 F	UC:66	8.1 ± 7.4 17.39 ± 14.2	ND
Pellino	2013	Prospective database	1990–2010	108	<70 (81) >70 (27)	29.9 ± 10.6 77.5 ± 3.1	32 M 49F 12 M 15F	UC: 108	8.9 ± 7.4 14.3 ± 8.1	Up to 3 years FU

Values expressed as mean ± SD

*ND* not documented, *UC* ulcerative colitis, *CD* Crohn’s disease, *FAP* familial adenomatous polyposis, *M* male, *F* female

^a^Number in bracket = standard error of mean

^b^Values expressed as median (range)

Patient age stratification was non-uniform across the papers. In addition, some studies considered longer-term follow-up. Therefore, in order to allow for data comparison, papers were considered as follows:

1. Patients were divided into a ‘younger’ and ‘older’ group with a cut-off age range of 50 ± 5 years. In studies where an age group spanned the cut-off range, i.e. 46–55, these patients were excluded from analysis [9, 13, 16]. Where data had been grouped into three or more comparative groups, the results were pooled into the two age categories in order to allow comparison of data. Results were weighted according to patient numbers in each age category. Nine papers satisfied the above criteria and were analysed together [1, 9, 10, 13, 16–20].
2. Separate sensitivity analysis was performed with a strict age cut-off of 50 years. Six papers were included [1, 10, 13, 16, 18, 20]. All compared outcomes for sections 1 and 2 were extracted at the 1-year (or earliest short-term) point.
3. Long-term outcomes (>10 years) were considered by three authors [13, 15, 16]. Data was extracted preferentially from the paper by Kiran et al. as follow-up stretched to 15 years. Additionally, in the paper by Delaney et al., it was difficult to determine the number of patients who were followed up for the entire 10-year period.
4. Two papers [2, 21] categorised ‘older’ patients as above the 50 ± 5 year cut-off. Therefore, they were considered separately alongside data from Ho et al. [10] and Delaney et al. [16] who had separate outcome data for patients >65 ± 5 years.

Table 2 displays the reported outcomes of interest for each paper.

**Table 2:** Outcomes of interest

Study	Year	Surgical complications	Bowel frequency	Incontinence	Pads/medication usage	Incontinence scoring system	Patient satisfaction	Quality of life	Sexual function	Anorectal physiology	Long-term outcomes	Age >65 subgroup
Pescatori	1990			X								
Lewis	1993		X	X								
Bauer	1996	X	X	X								
Dayton	1996	X	X	X						X		
Tan	1997	X		X	X	X						X
Takao	1998		X			X	X			X		
Delaney	2003	X	X	X			X	X	X		X	X
Chapman	2005	X	X	X				X	X		X	
Sun Ho	2005	X	X	X								X
Kiran	2009		X	X	X			X	X		X	
Pinto^a^	2009	X	X	X								X
Pellino^a^	2013	X	X	X	X			X			X	X

^a^Papers included only in 65 ± 5 analysis

### A comparative analysis of patients with age cut-off of 50 ± 5 years

There were 4327 versus 513 patients across the nine included papers. Table 3 describes the operative details for each study. Table 4 gives the age grouping breakdown from each paper.

**Table 3:** Operative details

Study	Year	Restorative proctocolectomy + IPAA (1 stage)	Completion proctectomy + IPAA (2 stage)	Ileostomy	Time to ileostomy closure	Pouch configuration	Stapled anastomosis?
Pescatori	1990	70 %	30 %	99.0 %	RP + IPAA: 24.7 weeks CP + IPAA: 150 weeks	J pouch 63 % S pouch 29 % W pouch 6 % L pouch 2 %	ND
Lewis	1993	100 %	0 %	ND	ND	J pouch 22.2 % W pouch 66.7 % None 11.1 %	ND
Bauer	1996	>50: 68.2 % <50: 53.7 %	>50: 31.8 % <50: 46.3 %	>50: 47.0 % <50: 57.4 %	ND	ND	0 % (all hand-sewn)
Dayton	1996	>55: 92.3 % <55: 83 %	>55: 8.7 % <55: 17 %	100 %	>8 weeks	J pouch 100 %	0 % (all hand-sewn)
Tan	1997	>50: 54 % <50: 40 %	>50: 46 % <50: 60 %	>50: 54 % <50: 55 %	ND	J pouch: >50: 96.4 %; <50: 90.45 % W pouch: >50: 3.57 %; <50 W 5.52 % S pouch: <50: 4.02 %	100 %
Takao	1998	100 %	0 %	100 %	8–12 weeks	ND	ND
Delaney	2003	>55: 74.0 % <45: 65 %	>55: 26.0 % <45: 35 %	>55: 92.4 % <45: 88 %	12 weeks	J pouch: >55: 91.3 %; <45 80 % S pouch: >55: 8.7 %; <45 20 %	>55: 86.2 % <45: 76 %
Chapman	2005	ND	ND	“Most”	12 weeks	J pouch 100 %	ND
Ho	2005	>50: 65.0 % <50: 58.1 %	>50: 33 % <50: 37.0 %	>50: 1.94 % <50: 4.8 %	>50: 13.8 weeks <50: 11.7 weeks	J pouch 100 %	ND
Kiran	2009	>55: 69.6 % <35: 59.3 %	>55: 30.4 % <35: 40.7 %	>55: 91.3 % <35: 89.4 %	ND	J pouch: 65.2 % >55; 58.3 % <55 S pouch: 34.8 % >55; 4.7 % <35	>55: 73.9 % <35: 56.1 %
Pinto	2009	>65: 75.7 % <65: 69.7 %	>65 24.3 % <65 30.3 %	100 %	ND	J pouch 100 %	>65: 87.9 % <65: 94.0 %
Pellino	2013	>70: 81.5 % <70: 85.2 %	>70: 18.5 % <70: 14.8 %	100 %	8–12 weeks	ND	>70: 66.6 % <70: 61.7 %

*ND* not documented, *RP* restorative proctocolecomy, *CP* completion proctectomy, *IPAA* ileal pouch-anal anastomosis

**Table 4:** Older and younger age groupings

Study	Age groups in study	Younger group (*N*)	Older group (*N*)
Takao^a^	<40 41–60^b^ >60	<40 (58)	>60 (17)
Pescatori	<45 >45	<45 (123)	>45 (33)
Dayton	<55 >55	<55 (423)	>55 (32)
Chapman^c^	<45 46–55^b^ >55	<45 (1688)	>55 (65)
Delaney^c, d, e^	<45 46-55^b^ 56-65 >65	<45(1323)	>55 (168)
Sun Ho^c, d^	<30 30-49 50-69 >70	<50 (227)	>50 (103)
Lewis^c^	<50 >50	<50 (18)	>50 (18)
Bauer^c^	<50 >50	<50 (326)	>50 (66)
Tan^c, d^	<50 >50	<50 (199)	>50 (28)
Total		4327	513

^a^Outcomes compared: anorectal physiology, patient satisfaction, incontinence scoring system, otherwise excluded in analysis

^b^Patient group excluded as spanning cut-off

^c^Studies included in sensitivity analysis

^d^Studies included in 65 ± 5 analysis

^e^Studies with >10 years follow-up

#### Surgical complications

Six papers [1, 10, 13, 16, 19, 20] discussed postoperative complications (Table 5). With the exception of small-bowel obstruction, which was significantly more common in younger patients (*p* = 0.034), there were no other differences identified in overall complication rates. There did however appear to be a trend towards increased rates of pouchitis in younger patients, although this did not reach statistical significance (*p* = 0.058). There was also a trend demonstrating towards a higher mortality rate in older patients (*p* = 0.070). Delaney et al. [16] showed a significantly higher pouch failure rate in those over 55 years of age at time of surgery (*p* < 0.000001); however, this result is not reflected in the overall analysis. Dayton et al. [19] documented that older patients were significantly more likely to be readmitted to hospital with dehydration (*p* = <0.01).

**Table 5:** Postoperative complications with age cut-off 50 ± 5 years

	No. of younger *N* (%)	No. of older *N* (%)	Total young	Total old	Chi-squared value	*p* value
Leak	65 (6.94)	7 (5.56)	937	126	0.336	0.562
Stenosis	72 (8.59)	20 (12.27)	838	163	2.212	0.137
Ischaemic bowel	3 (1.60)	1 (3.57)	188	28	0.523	0.469
Ischaemic pouch	2 (1.06)	1 (3.57)	188	28	1.119	0.290
Abscess formation	39 (9.40)	9 (6.87)	415	131	0.793	0.373
Cutaneous fistula	26 (6.27)	4 (3.05)	415	131	1.978	0.160
Vaginal fistula	11 (2.65)	3 (2.29)	415	131	0.052	0.820
Other fistula	4 (0.96)	0 (0)	415	131	1.272	0.259
Small bowel obstruction	143 (17.06)	17 (10.43)	838	163	4.473	*0.034*
Pouch excision/failure	210 (5.47)	22 (4.80)	3839	458	0.238	0.626
Pouchitis	172 (20.53)	23 (14.11)	838	163	3.580	0.058
Mortality	2 (0.17)	2 (0.87)	1164	229	3.289	0.070

Numbers in italics are statistically significant at *P* < 0.05

##### Functional outcomes

Functional outcomes were considered in all included papers. Due to the heterogeneity of reporting methods used, overall statistical comparisons have been made where data presentation has allowed.

##### Mean number of bowel motions/24 h

Four studies reported the mean bowel movements per 24 h [1, 10, 18, 19]. Mean weighted bowel frequency per 24 h was 5.55 ± 1.48 versus 6.79 ± 3.39 motions in the younger (*n* = 994) and older (*n* = 219) groups (unpaired *t* test, *p* < 0.0001).

##### Daytime and nocturnal bowel motions

Three papers considered bowel frequency as day and night motions [1, 13, 16]. Additionally, Dayton et al. [19] gave a separate nocturnal bowel motion frequency. The weighted mean daytime bowel frequency was 5.88 versus 6.06 in the younger (*n* = 1649) and older groups (*n* = 299). Nocturnal bowel frequency was 1.26 versus 1.96 in younger (*n* = 2072) and older (*n* = 331) groups.

##### Faecal incontinence

Incontinence episodes were considered by all included studies. However, again, heterogeneity in reporting methods meant it was impossible to combine data fully across all studies. Therefore, we have considered incontinence as follows: 1. Perfect/near perfect continence rates; 2. Daytime and night-time incontinence rates; 3. Type of incontinence.

Four studies [1, 16–18] gave indication of perfect or near-perfect continence rates. Overall, 74.75 % of the younger patients (*n* = 1353) versus 55.09 % of older patients (*n* = 285) experienced perfect or near-perfect continence (*χ*^2^ = 51.108 1df; *p* < 0.0001).

Four studies considered daytime and night-time incontinence rates [1, 13, 19, 20].

Dayton et al. [19] discusses frequencies as never/occasional/often/daily. Therefore, often/daily were combined to give a comparative daytime and night-time incontinence rate. Daytime incontinence rates were significantly higher in the older group (13.95 versus 5.56 %, *χ*^2^ = 18.352, *p* < 0.0001), as were night-time incontinence rates (29.65 versus 12.53 %, *χ*^2^ = 38.624, *p* < 0.0001).

Delaney et al. [16] discuss nocturnal seepage. At 1 year following surgery, 34 % of younger versus 49 % of older patients experienced nocturnal seepage (*χ*^2^ = 13.583, p = 0.0002).

The type of soiling encountered was discussed in four studies [10, 17, 18, 20]. Overall, 7.37 % of younger versus 11.04 % of older patients reported incontinence to mucus or flatus only (*χ*^2^ 1.9277, *p* = 0.165), whereas 4.19 % younger versus 5.06 % of older patients reported incontinence to faeces (*χ*^2^ 0.2283, *p* = 0.633).

##### Incontinence scoring system

The usage of incontinence scoring systems in reporting results was scarce, with only Takao et al. [9] employing the Cleveland Clinic Incontinence Scoring System and Tan et al. [20] using a different 12-point scoring system to report functional results. Neither author identified a significant difference in scores between the younger and older age groups.

##### Pad and medication usage

The usage of pads and medications was not frequently reported, and heterogeneity of reported data meant no combination of data was possible.

Dayton et al. [19] reported no significant difference in medication usage between older and younger patients.

Tan et al. report a daytime pad usage of 2.75 versus 16.7 % in younger and older groups (*χ*^2^ = 5.10, *p* = 0.024), and a night-time pad usage of 8.25 versus 16.7 % (*p* = 0.389) in older and younger groups. Medication usage was 33 versus 41.7 % (*p* = 0.323) in younger and older groups.

##### Other markers of function

Two papers [18, 20] discussed the ability to discriminate between flatus and faeces. Overall, 15.75 % younger versus 23.81 % older patients were unable to discriminate, but this did not reach statistical significance (*p* = 0.3409). Lewis et al. [18] found that 14/18 older vs 17/18 younger patients were ability to defer defecation for >15 min.

##### Patient satisfaction and quality of life data

Three papers considered patient satisfaction and/or quality of life data [9, 13, 16].

##### Patient satisfaction with surgery

Takao discusses patient satisfaction, describing patients as either ‘worse’, ‘no change’ or ‘improved’. In those >60, 12 patients (71 %) reported an improvement in satisfaction postoperatively, and 5 (29 %) reported no change. In the younger age group, <40, 44 patients (76 %) reported improvement, 12 patients (21 %) reported no change and 2 patients (3 %) reported worsening.

Delaney et al. [16] reported patient satisfaction with outcome of surgery as a mean score out of 10; at 1 year, this was 9.1 in the younger (<45) age group and 8.32 in the older (>55) age group.

##### Quality of life

Delaney et al. use the Cleveland global quality of life score (CGQL) in order to discuss differences in QoL between age groups. Patients under than 45 years of age tended to have a better overall score compared with older patients at 1, 3 and 5 years post surgery.

Chapman et al. [13] examined differences in restrictions between age groups. There were no significant differences detected in sexual, work, social or family activities noted between those <45 and those >45 at follow-up, with the exception of sexual function beyond 5 years which was significantly worsened in the >55 age category.

Seventy percent of patients >55 reported improved or not affected social activities following surgery; 84 and 82 % reported that undergoing IPAA had improved or not affected work and family life.

##### Anorectal physiology

Anorectal physiology was compared in three studies [9, 18, 19]. Differences in reporting data made overall statistical analysis difficult. Dayton et al. found that preoperative resting and squeeze pressures were significantly lower in patients >55 years; however, there were no significant differences postoperatively in these values between age groups. Similarly, Takao et al. and Lewis et al. found no significant differences in resting and squeeze pressures pre- and postoperatively between different age groups.

### Sensitivity analysis

Sensitivity analysis was performed on comparable functional outcome data with a strict age cut-off of 50 years, further excluding two papers [17, 19]. The total numbers included in sensitivity analysis were 3781 versus 448 patients in the older versus younger groups. Mean bowel actions per 24 h were 5.36 ± 2.48 versus 6.83 ± 3.90 motions in younger (*n* = 571) and older (*n* = 187) groups (unpaired *t* test *p* < 0.0001).

The difference in rates of near/perfect incontinence between older and younger groups remained highly significant (75.58 versus 55.16 %, *χ*^2^ = 47.658, *p* < 0.0001), as did the daytime (*p* < 0.0001) and night-time (*p* < 0.0001) incontinence rates. No differences were detected in the type of soilage experienced.

### Age 65 ± 5 years

Two additional papers [2, 21] considered a more elderly population, with Pellino et al. using age 70 as a cut-off and Pinto et al. using age 65. These papers were considered alongside patient subgroups from Delaney et al. (>65, *n* = 39), Sun Ho et al. (>70, *n* = 17) and Tan et al. (>70, *n* = 5) with patients divided into older (*n* = 104) and younger (*n* = 1981) categories.

Complication rates, functional outcomes and quality of life were compared between groups.

#### Complication rates

Complication rates were compared across three papers [2, 16, 21]. Table 6 gives the results. With the exception of dehydration and electrolyte imbalance (23.68 versus 60 %, *p* < 0.0001), there were no significant differences seen in postoperative complication rates or mortality between groups.

**Table 6:** Postoperative complications with age cut-off of 65 ± 5 years

	No. of younger *N* (%)	No. of older *N* (%)	Total young	Total old	Chi-squared value	*p* value
Leak	5 (4.39)	1 (1.67)	114	60	0.247	0.619
Stenosis	22 (5.15)	5 (1.17)	427	77	0.043	0.8366
Vaginal fistula	10 (2.54)	0 (0)	394	44	0.288	0.5913
Small bowel obstruction	46 (10.77)	6 (7.79)	427	77	0.346	0.5566
Pouch excision/failure	133 (6.36)	4 (4.49)	2092	89	0.237	0.6266
Pouchitis	50 (11.71)	9 (11.69)	427	77	0.000	0.9957
Mortality	1 (0.23)	0 (0)	427	77	0.181	0.6708
Medical complications						
Dehydration	27 (23.68)	36 (60)	114	60	20.901	*0.0001*
UTI	4 (3.51)	3 (5)	114	60	0.005	0.9442
MI	1 (0.877)	1 (1.67)	114	60	0.216	0.6424
LRTI	6 (5.26)	5 (8.33)	114	60	0.215	0.6431
PE	3 (2.63)	1 (1.67)	114	60	0.163	0.6864

*UTI* urinary tract infection, *MI* myocardial infarction, *LRTI* lower respiratory tract infection, *PE* pulmonary embolus

Numbers in italics are statistically significant at *P* < 0.05

#### Functional outcomes

The mean number of bowel motions per 24 h was 6.23 versus 6.50 in the younger and older groups [2, 10, 21]. 77.99 % of younger compared with 66.30 % of older patients had perfect continence (*χ*^2^ = 6.265, *p* = 0.0123) [10, 16, 21]. Pellino et al. [2] did not find a significant difference in either daytime or night-time incontinence rates between older and younger patients; however, they did report a statistically significant increase in the use of anti-diarrhoeal agents in older patients (*p* = 0.03).

#### Quality of life

Two papers [2, 16] considered quality of life following surgery. Unfortunately, both papers used different scoring systems therefore results could not be amalgamated. Pellino et al. found no significant difference in Inflammatory Bowel Disease Questionnaire (IBDQ) results between the older and younger patients. The quality of life data from Delaney and colleagues has been discussed previously.

### Long-term outcomes

Long-term outcomes (>10 years) were compared in three studies [13, 15, 16]. The data from Kiran et al. was used preferentially over that given by Delaney et al. due to the longer follow-up and completeness of long-term follow-up.

Unfortunately, due to the heterogeneity in methods of reporting functional outcomes in these papers, it was not possible to perform statistical analysis.

Kiran et al. [15] followed up 189 patients <35 years, 184 patients aged 35–55 years and 23 patients >55 years. Overall, there was an increase in the incidence of incontinence and urgency across all age groups during the 15 years of follow-up, with increased pad usage. However, patient satisfaction and quality of life data overall was stable over the 15-year time-frame, with no significant differences between the age groups. Mean stool frequency declined in the younger patients, but remained stable in the older patients, whereas nocturnal stool frequency increased significantly in younger patients. Daytime seepage increased significantly in older patients, and for those aged 35–55 years nocturnally.

Chapman et al. had 10-year follow-up data available for 892 patients <45 years, 118 patients 45–55 years and 25 patients >55 years of age. They reported that whilst patients >55 suffered significantly more daytime and night-time incontinence at 1 and 3 years compared to those <45, this was no longer apparent at 5 and 10 years follow-up, with no significant difference in incontinence rates between younger and older groups. Patients >55 years had a daytime incontinence rate of 15.2 % at 1 year, and 12 % at 10 years, and those <45 years had a daytime incontinence rate of 4.1 % at 1 year and 4.8 % at 10 years. Night-time incontinence rates in those >55 years were 26.1 % at 1 year and 24 % at 10 years and 9.4 % at 1 year and 12.2 % at 10 years in those <45 years.

With the exception of sexual restrictions, which was significantly more common in those older than 55 years at 5 and 10 years (3.1 versus 21.7 % at 10 years follow-up, *p* < 0.01), there was no significant difference in other life areas such as work, travel, social, family relations and sport.

## Discussion

The incidence of inflammatory bowel disease in the elderly (>60 years) is currently 10–15 % [22], with the majority presenting in their 60s; however, 25 % present in their seventies and a further 10 % in their eighties [23]. With improving healthcare delivery meaning people live for longer, these figures are set to increase.

Medical treatment in ulcerative colitis is similar across all age groups, with similar response rates to treatment [24]. Failure of medical treatment remains the most common reason for proceeding to surgical intervention; however, the presence of dysplasia is another common indication amongst this patient population [25], with Bauer et al. demonstrating a significantly higher rate of dysplasia and malignancy in older patients [1]. A recent meta-analysis found that older patients with ulcerative colitis were no more likely to undergo surgery when compared to younger patients [26], which is in the region of a third of all cases [4]. This is in spite of the fact that disease severity tends to be less in the older population [23].

The undertaking of major reconstructive surgery in the form of ileoanal pouch creation following proctocolectomy in older patients has been a fairly contentious issue. Initially, it was believed that due to the high incidence of complications, with around a 20 % morbidity [27], that restorative proctocolectomy should not be performed in patients older than 50. Instead patients were left with an end ileostomy, which is in itself associated with a relatively high morbidity, although several studies have demonstrated a similar quality of life when compared to the general population [28, 29]. More recent studies, including those in this review have advised that IPAA can be safely performed in older patients, even those in their 70s or 80s [30, 31]. Guidance published by the American Society of Colon and Rectal Surgeons recommend that chronological age should not be a reason in itself to deny IPAA in older patients [32].

This analysis supports the view that in terms of safety, IPAA in patients over 50 is feasible with comparable complication rates to those seen in younger patients. A recent multicentre registry analysis undertaken by Cohan and colleagues [4] which included 2493 patients undergoing IPAA, with 254 above the age of 60, found that in terms of postoperative complications rates, there was no significant increase in older patients; however, there was an increased length of stay in the older group.

Small-bowel obstruction was found to be significantly more common in younger patients in this review. The reasons as to why this is remain unclear; however, this difference was not replicated in the separate analysis of patients over 65. Otherwise, the only other difference worthy of note was the significantly increased risk of dehydration and electrolyte imbalance (23.68 versus 60 % in those over 65). This was in the context of loop ileostomy formation in the early postoperative phase. Several studies have shown dehydration to be the most common cause of patient readmission following ileostomy creation [33, 34]. Paquette et al. [35] found age >50 to be an independent factor for a readmission with renal failure following ileostomy creation, with IPAA associated with readmission for dehydration in their series of 201 patients. Overall, they found that 30-day readmission rates with dehydration following loop ileostomy creation were in the region of 25 %. Sixteen percent of those >50 versus 5 % of those <50 were admitted within 30-days with renal failure secondary to ileostomy. The rate of renal failure with IPAA was 8 % in >50 versus 7 % in <50. The reasons as to why the rates of dehydration and electrolyte imbalance in the papers included in the review are much higher are unclear; this may be secondary to these patients being at the extremes of age i.e. >65 years old, where there is more likely to be a degree of pre-existing renal impairment or the concomitant use of diuretic agents.

With regards to functional outcomes, there was a significant difference noted in terms of incontinence rates, with older patients significantly more likely to suffer incontinence, both during the day and nocturnally. The number of bowel motions per 24 h was statistically higher in those over 50 years of age; however, the likely impact of this on overall daily living is likely to be minimal, with an average of only one extra visit to the toilet in 24 h. Functional results were seen to decline across all age groups over time. In both of the papers where long-term functional data was analysed, the significantly different incontinence rates between older and younger groups seen at 1 year were diminished in the longer term.

The authors Pellino et al. have published a further paper discussing outcomes with patients over the age of 80 years [31]. Although at 6 months older patients had more nocturnal seepage, anti-diarrhoeal usage and a trend towards higher daytime incontinence rates, these issues were largely resolved by 12 months, with only nocturnal seepage more common in the elderly patient group. Most importantly, all older patients were satisfied with their outcomes and would undergo surgery again. Patient satisfaction and perceived quality of life following surgery are arguably the most important aspect of this review, as beyond ensuring comparable outcomes in terms of safety, the acceptability of results to patients is of utmost importance. Unfortunately, only three papers considered this outcome; however, these showed that overall patient satisfaction and quality of life were maintained following surgery in older patients, with the exception of Delaney et al. [16], who did report a significant difference in CGQL scores in older patients at 1, 3 and 5 years following surgery. Happiness levels were comparable across all ages. These positive outcomes with respect to satisfaction with surgery and QoL are in spite of the statistically significant increase in day and night incontinence rates.

Overall, the outcomes shown demonstrate that the consideration of RPC in older patients is warranted, with acceptable complication rates not dissimilar to those seen in younger patients. Careful fluid balance must however be achieved to avoid dehydration, in particular when older patients have a temporary loop ileostomy formed as this predisposes them to a significantly higher risk of electrolyte imbalance and dehydration. Additionally, the operating surgeon should ensure that there is at least 200 cm of small bowel proximal to the site of stoma creation in order to reduce this risk further. A shorter segment of proximal small bowel will also predispose the patient to a greater risk of incontinence, faecal urgency, seepage and looser stool once the stoma is reversed. Older patients should also be counselled as to the higher likelihood of faecal incontinence and nocturnal seepage following stoma reversal.

Further studies should include age-related analysis regarding postoperative quality of life and satisfaction with surgery, as these aspects are fundamental in the decision to recommend this major surgery to older patients.

As with all surgery, thorough patient optimisation and careful case selection are vital, with consideration given to the suitability of each case on its own merit; age as a number alone is insufficient as a basis for decision regarding the type of surgery performed. Patients should receive adequate support with regards to management of any functional deterioration in order to maintain high levels of patient satisfaction and acceptable postoperative quality of life.

### Limitations

The main limitations of this review are related to the heterogeneity of presented data which at times made statistical comparison impossible. Furthermore, the majority of the papers grouped patients according to age alone, with differing disease pathology and operative techniques. Unfortunately, data was not presented in such a way to allow comparison between age groups for separate disease states. Included papers were also in the most part either retrospective or involved retrospective analysis of a prospectively maintained database. In addition, different age cut-offs were employed to define older and younger groups between papers, although sensitivity analysis demonstrated extremely similar outcomes to analysis across all studies.
